# Shape Optimization and Experimental Investigation of Glue-Laminated Timber Beams

**DOI:** 10.3390/ma17246263

**Published:** 2024-12-21

**Authors:** Paweł Szeptyński, Dorota Jasińska, Leszek Mikulski

**Affiliations:** Division of Structural Mechanics and Material Mechanics, Faculty of Civil Engineering, Cracow University of Technology, ul. Warszawska 24, 31-155 Kraków, Poland; dorota.jasinska@pk.edu.pl (D.J.); leszek.mikulski@pk.edu.pl (L.M.)

**Keywords:** structural optimization, optimal shaping, glue-laminated timber, control theory, experimental investigation

## Abstract

This study investigated the optimal shape of glue-laminated timber beams using an analytical model of a slender beam, taking into account the anisotropy of its strength properties as well as boundary conditions at the oblique bottom face of the beam. A control theory problem was formulated in order to optimize the shape of the modeled beam. Two optimization tasks were considered: minimizing material usage ($V_{min}$) for a fixed load-carrying capacity (LCC) of the beam and maximizing load-bearing capacity ($Q_{max}$) for a given volume of the beam. The optimal solution was found using Pontryagin’s maximum principle (PMP). Optimal shapes were determined using Dircol v. 2.1 software and then adjusted according to a 3D finite element analysis (FEA) performed in Abaqus. The final shapes obtained through this procedure were used in the CNC-based production of three types of nine beams: three reference rectangular beams, three $V_{min}$ beams, and three $Q_{max}$ beams. All specimens were subjected to a four-point bending test. The experimental results were contrasted with theoretical assumptions. Optimization reduced material usage by ca. 12.9% while preserving approximately the same LCC. The maximization of LCC was found to be rather unsuccessful due to the significant dependence of the beams’ response on the highly variable mechanical properties of GLT.

## 1. Introduction

The popularity of glue-laminated timber (GLT) has steadily increased since its first significant use in 1893 [1]. This growth has been driven by several factors, including its environmental friendliness [2,3], significant carbon storage capacity [4], economic efficiency [5], renewability [6], aesthetics, and impressive load capacity-to-weight ratio. Additionally, GLT offers exceptional versatility in shaping structures and structural elements, providing an opportunity for optimization.

The optimization of timber structures is the subject of ongoing research. A variety of different optimization methods have been employed in order to minimize either the material consumption or the total cost of constructing a timber structure. Šilih et al. minimized the cost of a timber truss through the use of mixed-integer nonlinear programming, optimizing the topology of the structure, the size of truss members, and the number of fasteners [7]. A similar analysis was performed by Kravanja and Žula concerning a timber hall structure with repetitive timber portal frames [8,9]. Topology optimization (TO) algorithms were employed by Gao et al. in [10] in strut-and-tie models in order to minimize the total material consumption of steel–timber structures. Hua et al. [11] presented an algorithm that enabled the construction of 3D timber framework shells reticulated with a Voronoi tessellation, accounting for the manufacturability of timber parts. In [12], Jelušič and Kravanja used multi-parametric mixed-integer nonlinear programming to optimize the cost of constructing two variants of a timber floor system (one with a single system of joists and a second with additional secondary joists) while taking into account code requirements. In [13], Villar-García et al. minimized the total cost of constructing a timber truss roof structure using genetic algorithms (GAs). A similar analysis using GAs was performed in [14] to minimize material usage in roofs made of double-tapered GLT beams. Decker et al. showed that particle swarm optimization is suitable for the multi-objective optimization of a timber building, taking into account heat, vibration, strength, and energy requirements [15]. Kaziolas et al. used simulated annealing (SA) and GAs to conduct a life cycle analysis and optimized the dimensions of timber structural elements for a frame structure [16].

In [17], which had a goal similar to that of the present study, Mayencourt and Mueller used a combination of the COBYLA algorithm, GAs, and direct search methods to optimize the shape of a mass timber beam described using NURBS surfaces.

Whole structures and structural elements are not the only factors considered in optimization. GLT and CLT, as structural materials with complex internal topology, may also be optimized to enhance their performance or reduce their mass or cost of production. De Vito et al. used TO to optimize the core of engineered wood products, such as GLT or cross-laminated timber (CLT) [18]. Mayencourt and Mueller [19] used nonlinear programming to optimize the layout of cavities in a five-ply CLT panel, reducing its mass by 18% without decreasing its performance compared to solid CLT. Pech et al. [20] used metaheuristic algorithms such as local search (LS), iterated local search (ILS), and GAs to optimize the layout of a wooden lamella with different mechanical properties on a GLT beam. As a result, the deflection of such a beam could be reduced by up to 15–20%.

Among multiple methods of structural optimization, the control theory approach, which is based on Pontryagin’s maximum principle, guarantees the satisfaction of the conditions necessary for optimality [21,22]. For simple structural systems, such as beams, arches, or trusses, it is possible to assign each specific cross-section a unique value of a single variable. This enables the formulation of a one-dimensional control theory problem, which may be solved using PMP. This approach was successful in the optimization of bar structures in which the cross-sections of bars [23,24,25,26,27,28], composite beams [29], or functionally graded beams [30] were optimized. The global layout of the structure may be optimized, e.g., by controlling the local curvature of the elastic arches [31]. Code requirements may be easily incorporated in the formulation of a control theory problem [25]. The thicknesses of circular and rectangular plates were also optimized using PMP-based methods [32,33]. The stability of elastic structures was investigated in [33,34,35].

The PMP-based optimization method was employed in solving the optimal design problems presented in this article for two reasons: First, Pontryagin’s maximum principle guarantees the satisfaction of the conditions necessary for optimality. These conditions were verified when solving the optimization problem with the use of Dircol software [36]. Second, commonly used optimization tools, such as the Tosca add-on to Abaqus or topology optimization in Ansys Mechanical, are not able to account for certain structural optimization features in civil engineering. Third, they cannot employ nonlinear limit state conditions for anisotropic solids; only a linear combination of given design responses may be involved in optimization constraints. Fourth, topology optimization can only be performed in the design zone, which is defined in advance. Specifically, this approach excludes from the design zone the boundaries on which support or load conditions are prescribed since no rule describes how these boundary conditions should propagate through a continuum, the shape of which changes in the course of optimization. While “freezing” the supported and loaded areas is a common approach in optimizing machine parts (due to assumed technology of connection), this approach cannot be used when designing prefabricated structural elements in civil engineering, such as simply supported beams, which are considered in this study. The limitations of the standard topology optimization approach make the abovementioned software unsuitable for these cases for practical reasons. On the other hand, the simplified PMP-based optimization of beams is flexible as it includes various types of supports and nonlinear inequality constraints. Since this approach is biased by oversimplifying assumptions, it is necessary to validate the obtained results using more accurate methods. For this reason, a 3D finite element method (FEM) model was developed, and a nonlinear analysis was carried out using Abaqus 2023 software [37]. This approach has only been employed theoretically so far; the novelty of this study is that the theoretical optimization results are verified experimentally.

The aim of this study was to experimentally verify the effectiveness of structural optimization via the control theory. The present research focuses on the optimal shaping of GLT beams, specifically addressing the design of a simply supported beam subjected to symmetric four-point bending. Simply supported beams are among the most commonly utilized prefabricated beam types. A span length of L=4 m was chosen. A constant thickness of b=25 cm was assumed in order to avoid lateral torsional buckling in the analyzed beams. For volume-minimizing optimal beam design, a reference total load of $Q_{ref}=170\;kN$ was selected and applied symmetrically as two concentrated forces spaced 1.334 m apart. Three optimization problems are considered in this study:

1. PROBLEM 1: Minimizing the volume of the beam for a given load $Q_{ref}=170\;kN$, assuming a constant height of the beam. The obtained minimal admissible volume is denoted by $V_{ref}$.
2. PROBLEM 2: Minimizing the volume $V_{min}$ of a beam of variable height for a given load $Q_{ref}$ (the same as in problem 1).
3. PROBLEM 3: Maximizing the load $Q_{max}$, which may be applied to a beam of variable height and given volume $V_{ref}$ (calculated in problem 1).

The first aim of this study was to determine the minimal reference volume of a beam of constant height (REF beam) and with a load-carrying capacity (LCC) of 170 kN. Having determined this volume, the problem of minimizing the required material volume was stated for a beam of variable height ($V_{min}$ beam) and the same LCC as the REF beam. Finally, another beam of variable height and fixed volume $V_{ref}$ ($Q_{max}$ beam) was optimized in order to maximize its LCC. Only the ultimate limit state (ULS) was considered in all optimization tasks; the serviceability limit state (SLS) was disregarded. All optimization tasks were solved within the framework of the control theory, employing PMP. A solution was obtained via the direct collocation method, which was implemented in Dircol software. To mathematically express the optimization problems in a manner suitable for PMP formalism, the Timoshenko–Ehrenfest beam theory was employed. This theory’s stress state predictions were adjusted to incorporate the boundary conditions on the oblique bottom face of the beam, a critical factor in designing beams of variable height. The considered model is one-dimensional, as required by control theory. Therefore, the obtained solutions were numerically validated via the finite element method, which was implemented in Abaqus software. A full 3D analysis revealed the need for some minor adjustments to the optimal shapes in order to satisfy the ULS condition. Finally, the optimally designed beams were manufactured from the GL24h GLT using computerized numerical control (CNC) technology. Three specimens of each beam type were produced. The number of specimens investigated was strongly constrained by the relatively high cost of producing these elements. These beams were tested in a laboratory, and the experimental results were compared to the numerical predictions.

Briefly, this article is divided into the following sections: Section 2 presents the materials under investigation; in particular, the physical and mechanical properties of GLT and elastomer spacers are provided. An energy-based limit state criterion for anisotropic solids exhibiting asymmetry in the elastic range is also presented. An analytical model of an anisotropic beam of variable height is discussed in detail in Section 3. Section 4 deals with the formulation of an optimization problem using the formalism of the control theory; specifically, governing equations, objective functions, and constraints are described. The results of PMP-based optimization are presented and validated via finite element analysis (FEA). Section 5 provides a detailed description of the experimental setup and procedures. The results of force–displacement and strain measurements are presented. A discussion of the results is given in Section 6. Finally, the conclusions and an overview of future research are presented in Section 7.

## 2. Materials

### 2.1. Glue-Laminated Timber

The material under consideration is the GL24h-class GLT. The mechanical properties of this material, according to [38], are listed in Table 1.

The compressive strength perpendicular to the grain in the area of the supports and in the region of the applied loads was increased by 75%, according to the recommendations of EN 1995-1:2010, Section 6.1.5, items (2) and (3) [40].

It should be emphasized that all strength properties are given as 5% percentile values, meaning that with a probability of 95%, the true values will be equal or larger. Similarly, most stiffness moduli are given as mean values. Moreover, timber classification is seldom performed according to statistical distributions obtained from multiple tests on a sample of a given population. The common practice is to classify the timber based on a visual evaluation of wood quality (the presence, concentration, and size of knots, twisting of the grain, etc.). For this reason, it is usually impossible for the designer to acquire precise information about the statistical distribution of timber stiffness and strength. As a result, true property values often differ considerably from those assumed in the design process according to class; this strongly affects the process of optimization, for which exact information on the true strength and stiffness is required.

Since such information is, in practice, unavailable to the designer, it was assumed that optimization is performed with values provided by the appropriate standard.

It should be noticed that EN 14080 [38] (similar to EN 338 [41] for softwood and hardwood) provides only basic information about the strength properties of timber, and no reference is made to the material’s resistance in complex (multiaxial) stress states. This problem is addressed in the design procedures presented, e.g., in [40], although only a number of specific cross-sectional force interactions are considered in that study. The goal of this research was to consider a general plane stress state. For this reason, an energy-based limit state criterion for orthotropic materials was employed [42]. According to this proposition, the limit state criterion takes the form of a combination of terms of the main decomposition of elastic strain energy [43], in which the contribution of each energy term is altered by a rational function [44] depending on the invariants of a corresponding eigenvector of elasticity tensors. As a result, the limit state condition may be represented by a multidimensional quadric surface, and the condition itself may be expressed in terms of the strength parameters of the material. In the case of an orthotropic material experiencing plane stress, the general form of the limit state condition is as follows:

(1) $$F(\sigma )=A_{I}\sigma_{I}^{2}+B_{I}\sigma_{I}+A_{II}\sigma_{II}^{2}+B_{II}\sigma_{II}+A_{III}\sigma_{III}^{2}=1$$

where

(2) $$\sigma_{I}=\sigma_{xx}\cos\kappa +\sigma_{zz}\sin\kappa ,\;\;\;\;\;\;\;\sigma_{II}=\sigma_{zz}\cos\kappa -\sigma_{xx}\sin\kappa ,\;\;\;\;\;\;\;\sigma_{III}=\tau_{xz}$$

(3) $$A_{I}=\frac{1}{f_{t,0,k}^{2}}\left[\frac{\sin^{2}\kappa }{K^{2}k_{2}\left(\sin^{4}\kappa -\cos^{4}\kappa \right)}-\frac{\cos^{2}\kappa }{k_{1}\left(\sin^{4}\kappa -\cos^{4}\kappa \right)}\right]$$

(4) $$A_{II}=\frac{1}{f_{t,0,k}^{2}}\left[\frac{\sin^{2}\kappa }{k_{1}\left(\sin^{4}\kappa -\cos^{4}\kappa \right)}-\frac{\cos^{2}\kappa }{K^{2}k_{2}\left(\sin^{4}\kappa -\cos^{4}\kappa \right)}\right]$$

(5) $$A_{III}=\frac{1}{f_{v,k}^{2}}$$

(6) $$B_{I}=\frac{1}{f_{t,0,1}}\left[\cos\kappa \frac{k_{1}-1}{k_{1}}+\frac{\sin\kappa }{K}\frac{k_{2}-1}{k_{2}}\right]$$

(7) $$B_{II}=\frac{1}{f_{t,0,1}}\left[\frac{\cos\kappa }{K}\frac{k_{2}-1}{k_{2}}-\sin\kappa \frac{k_{1}-1}{k_{1}}\right]$$

(8) $$K=\frac{f_{t,90,k}}{f_{t,0,k}},\;\;k_{1}=\frac{f_{c,0,k}}{f_{t,0,k}},\;\;k_{2}=\frac{f_{c,90,k}}{f_{t,90,k}}$$

Parameter κ is a function of the stiffness distributor, an invariant of the eigenvectors of elasticity tensors, determining their form as well as the form of the main decomposition of elastic strain energy [45]. For the case of plane orthotropy the κ parameter can be expressed as follows:

(9) $$\kappa =-\mathrm{atan}\left[\frac{E_{0,m}}{2\nu }\left(\frac{1}{E_{90,m}}-\frac{1}{E_{0,m}}-\sqrt{{\left(\frac{1}{E_{90,m}}-\frac{1}{E_{0,m}}\right)}^{2}+\frac{4\nu^{2}}{E_{0,m}^{2}}}\right)\right]$$

The parameters of the limit state criterion given in Equation (1) for the GL24h-class GLT are listed in Table 2.

**Figure 1:**
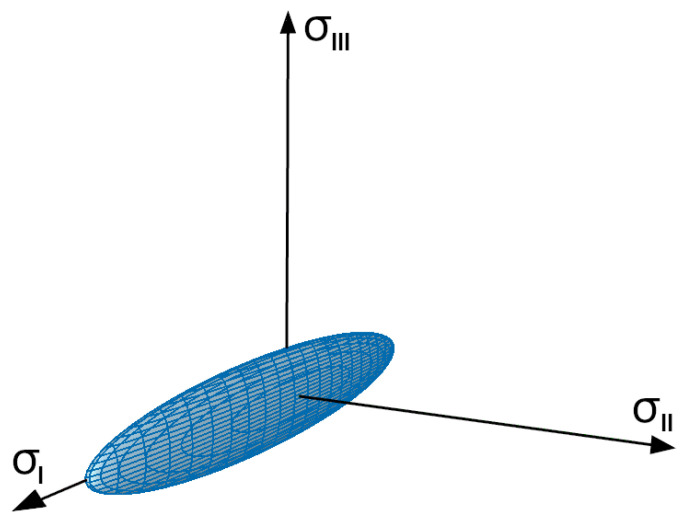
Limit state surface given in Equation (1). Axis scales are equal.

It can be confirmed via direct calculation (see, e.g., [46]) that the quadric surface described by Equation (1), with the parameters given in Table 2, is an ellipsoid; this is a closed limit state surface that corresponds well with the mechanical characteristics of wood, for which there are no so-called safe stress states [47]. A plot of the limit state surface is given in Figure 1.

### 2.2. Polymer Spacers

**Figure 2:**
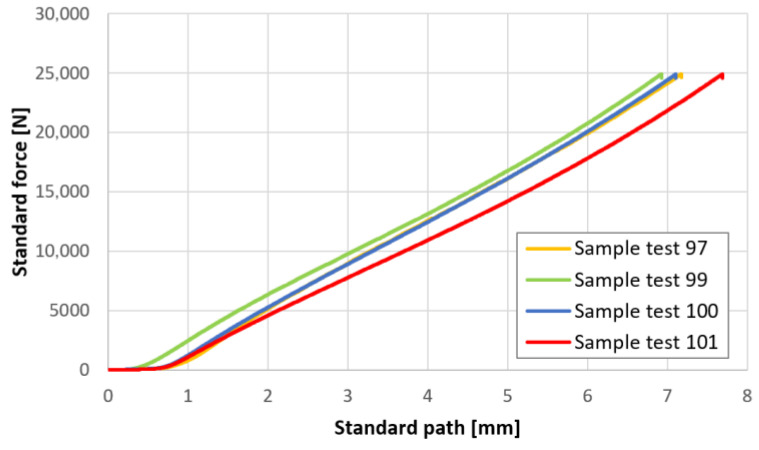
Force–displacement curves for uniaxial compression test on polymer spacer samples.

To avoid stress concentration in the area of supports and applied loads in the designed beams, polymer spacers measuring 147 mm in length, with a thickness of 15 mm, were placed directly between the beam and the steel plates on which the optimized beam was placed as well as between the beam and the bearings through which the load is transmitted. Since these spacers are much more flexible than GLT and steel, their presence influences the deflection measurement. To estimate this error, uniaxial compression tests were carried out on four specimens with the use of a Zwick/Roell 1455 Universal Testing Machine. The maximum force range was 0–24 kN with a resolution of 0.1 kN, while the resolution of the extensometer was 0.005 mm. Test Expert III software was used. Standard force vs. standard path curves are presented in Figure 2.

The initial parts indicating negligible stiffness are due to the unevenness of the top and bottom faces of the specimens. These sections were disregarded in the evaluation of linear regression when estimating the longitudinal stiffness of the material. The polymer’s Young’s modulus at compression was approximately $E_{sp}=22.82\;MPa$.

## 3. Analytical Model of Beam

### 3.1. Geometry and Static Scheme of Designed Beams

**Figure 3:**
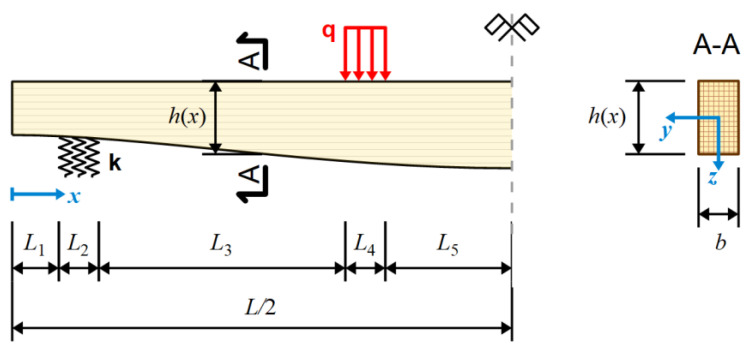
Static scheme of optimized beam (dimensions not to scale).

A simply supported beam of variable height $h\left(x\right)$ subjected to four-point bending was considered. A constant width of b=25 cm was assumed, while height distribution was the subject of an optimization task discussed in the following section. The span length was $L_{e}=400\;cm$, which, together with 40 cm overhangs, gave a total beam length of L=480 cm. Supports, together with polymer spacers, were modeled as sections of beams lying on a Winkler-type elastic foundation. The load, which was also applied through the elastic spacers, was modeled as a uniformly distributed load. This is simply an approximate approach; however, the presence of steel bearings and polymer spacers provides a more or less uniform distribution of contact stresses, justifying such a simplification. Due to the symmetry of the problem, only half of the beam was modeled. A static scheme of the beam is presented in Figure 3. The lengths of characteristic sections are as follows:

(10) $$L_{1}=32.65\;cm,\;\;\;\;\;\;\;\;\;L_{2}=14.7\;cm,\;\;\;\;\;\;\;\;\;L_{3}=118.6\;cm,\;L_{4}=14.7\;cm,\;\;\;\;\;\;\;\;\;L_{5}=59.35\;cm$$

### 3.2. Analytical Model

The Timoshenko–Ehrenfest beam theory was employed to model the analyzed beam. A system of governing equations for a beam with a rectangular cross-section b×h, where h=h(x), may be written as follows:

(11) $$\left\{\begin{array}{l} \frac{dw}{dx}=\phi (x)+\frac{Q(x)}{\kappa Gbh(x)}\\ \frac{d\phi }{dx}=-\frac{12M(x)}{Eb{\left[h\left(x\right)\right]}^{3}}\\ \frac{dM}{dx}=Q(x)\\ \frac{dQ}{dx}=-q\left(x\right)+k\left(x\right)bw\left(x\right)-\gamma bh\left(x\right) \end{array}\right.$$

where

(12) $$k\left(x\right)=\left\{\begin{array}{cc} k_{sp}=const. & \Leftrightarrow x\in \left(L_{1};L_{1}+L_{2}\right)\\ 0 & \mathrm{otherwise} \end{array}\right.$$

(13) $$q\left(x\right)=\left\{\begin{array}{cc} q_{P}=const. & \Leftrightarrow x\in \left(L_{1}+L_{2}+L_{3};L_{1}+L_{2}+L_{3}+L_{4}\right)\\ 0 & \mathrm{otherwise} \end{array}\right.$$

Young’s modulus E is taken as $E_{0,m}$, while the modulus of rigidity G is taken as $G_{m}$. The shear correction factor κ for the rectangular cross-section is κ=5/6. Winkler’s subgrade modulus $k_{s}$ is estimated by assuming that the elastic spacer of thickness $h_{sp}=15\;mm$ undergoes simple compression only while the steel plate, roller, and bearing are rigid, such that $k_{sp}=\frac{E_{sp}}{h_{sp}}=1520\;N/m^{3}$. The external load $q_{P}$ is assumed to be equal for the reference beam and the $V_{min}$ beam; $q_{P}=578\;kN/m$. The density of the GLT 24h is taken as $\rho =420\;kg/m^{3}$, according to EN 14080:2013, which determines the specific weight γ of the GLT. For the $Q_{max}$ beam, $q_{P}$ is unknown and is determined via the optimization process as the largest permissible load for a given volume of GLT.

In the Timoshenko–Ehrenfest beam theory, which describes first-order shear deformation, any plane cross-section (corresponding to the variable *x*) perpendicular to the undeformed beam’s axis is assumed to undergo rigid translation w(x) and rigid rotation ϕ(x). As a result, the distribution of axial normal stresses through the beam’s thickness is linear:

(14) $$\sigma_{xx}\left(x,z\right)=\frac{12M(x)}{b{\left[h\left(x\right)\right]}^{3}}z$$

According to the equilibrium equation (after neglecting the body forces),

(15) $$\left\{\begin{array}{c} \frac{\partial \sigma_{xx}}{\partial x}+\frac{\partial \tau_{xz}}{\partial z}=0\\ \frac{\partial \tau_{xz}}{\partial x}+\frac{\partial \sigma_{zz}}{\partial z}=0 \end{array}\right.$$

The through-the-thickness distribution of shear stress $\tau_{xz}$ in a symmetric cross-section must be given by a symmetric polynomial function of the second degree. However, during analysis [29], it was noted that the obliquity of the beam’s bottom face introduces a complex stress state in the bottom fibers that is decisive in optimizing the beam. A symmetric quadratic function is not suitable for describing the shear stress distribution when the horizontal top face is traction-free and the bottom face is inclined. In fact, simple beam theories, such as the Bernoulli–Euler and Timoshenko–Ehrenfest theories, assume that the beam is prismatic. Analyzing beams with variable height within these theoretical frameworks is formally incorrect. On the other hand, the errors introduced in such an analysis are often negligible, especially when the slope of the top or bottom face is not large. For this reason, the symmetric quadratic distribution of $\tau_{xz}$ was extended with a skew-symmetric linear term, with which it was possible to account for the boundary conditions prescribed for the top and bottom faces. It should be noted, however, that in this case, the equilibrium Equation (15)_1_ is formally violated. The modified distribution is given by the following function:

(16) $$\tau_{xz}\left(x,z\right)=D_{0}+D_{1}z+D_{2}z^{2}$$

Coefficient $D_{2}$ is determined according to Equation (15)_1_, while the other two coefficients are obtained by assuming that $\tau_{xz}\left(x,\frac{h}{2}\right)=\tau_{n}(x)$ and $\tau_{xz}\left(x,-\frac{h}{2}\right)=0$, where $\tau_{n}$ is the horizontal shear component of the stress state in the bottom fibers.

**Figure 4:**
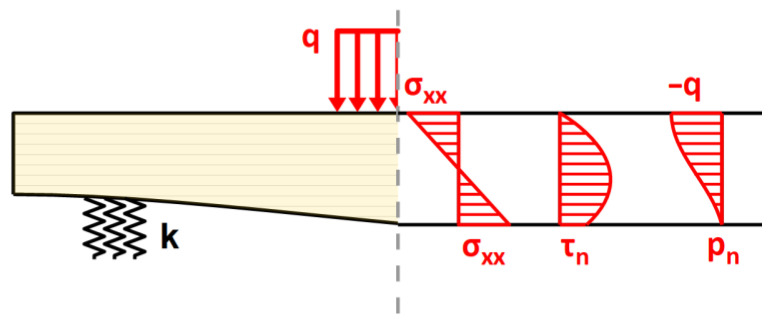
Through-the-thickness distribution of stress tensor components.

Since the distribution of $\tau_{xz}$ is quadratic with respect to z, according to equilibrium Equation (15)_2_, the through-the-thickness distribution of the transverse normal stress $\sigma_{zz}$ is given by a cubic function (see Figure 4) of the following general form:

(17) $$\sigma_{zz}\left(x,z\right)=C_{0}+C_{1}z+C_{2}z^{2}+C_{3}z^{3}$$

**Figure 5:**
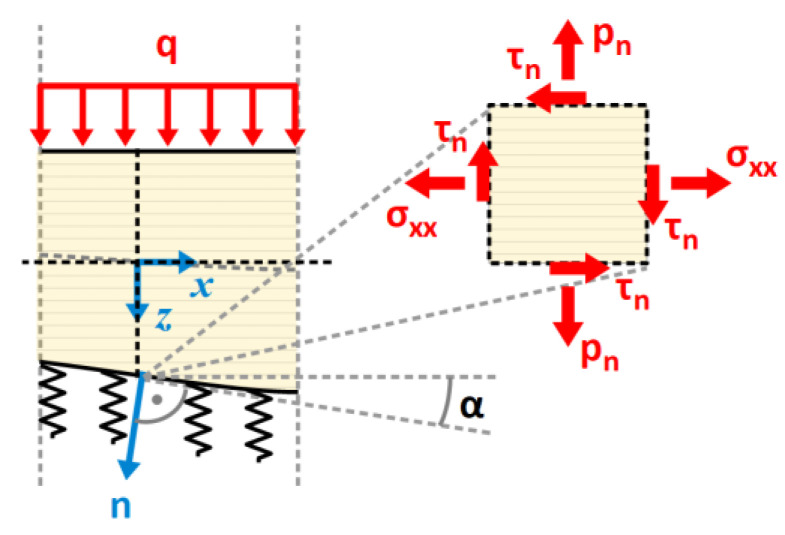
Stress state at the oblique bottom face of the beam.

Coefficients $C_{0},\ldots ,\;C_{3}$ are determined with the use of a linear system of four equations, namely, equilibrium Equations (15)_2_, which are written for the top and bottom faces, as well as the static boundary conditions $\sigma_{zz}\left(x,\frac{h}{2}\right)=p_{n}(x)$ and $\sigma_{zz}\left(x,-\frac{h}{2}\right)=-q(x)$, where $p_{n}$ is the vertical normal component of the stress state in the bottom fibers. Stresses $p_{n}$ and $\tau_{n}$ may be found from the boundary conditions at the oblique bottom face (Figure 5):

(18) $$\left[\begin{array}{cc} \sigma_{xx}\left(x,\frac{h}{2}\right) & \tau_{n}(x)\\ \tau_{n}(x) & p_{n}(x) \end{array}\right]\left[\begin{array}{c} -\sin\left[\alpha (x)\right]\\ \cos\left[\alpha (x)\right] \end{array}\right]=\left[\begin{array}{c} 0\\ -k(x)w(x) \end{array}\right]$$

where α is the angle of inclination of the bottom face to the horizontal plane. Then, the following equations are obtained:

(19) $$\tau_{n}\left(x\right)=\sigma_{xx}\left(\frac{h}{2}\right)tg\;\alpha =\frac{6M\left(x\right)}{b{\left[h\left(x\right)\right]}^{2}}\frac{dh}{dx}$$

(20) $$p_{n}\left(x\right)=\tau_{n}tg\;\alpha -kw\sec\alpha =\frac{6M\left(x\right)}{b{\left[h\left(x\right)\right]}^{2}}{\left(\frac{dh}{dx}\right)}^{2}-k\left(x\right)w\left(x\right)\sqrt{1+{\left(\frac{dh}{dx}\right)}^{2}}$$

Finally, we can express the coefficients in Equations (16) and (17)—which are in fact functions of the chosen cross-section, identified by *x*—in the following way:

(21) $$D_{0}\left(x\right)=\frac{1}{4}\left(2\tau_{n}\left(x\right)-D_{2}\left(x\right){\left[h\left(x\right)\right]}^{2}\right)$$

(22) $$D_{1}=\frac{\tau_{n}(x)}{h(x)}$$

(23) $$D_{2}\left(x\right)=\frac{6}{b}\left[\frac{3M\left(x\right)}{{\left[h\left(x\right)\right]}^{4}}\frac{dh}{dx}-\frac{Q\left(x\right)}{{\left[h\left(x\right)\right]}^{3}}\right]$$

(24) $$C_{0}\left(x\right)=\frac{-4q\left(x\right)+4p_{n}\left(x\right)+\left(\partial \tau_{b}\left(x\right)-\partial \tau_{t}\left(x\right)\right)h\left(x\right)}{8}$$

(25) $$C_{1}\left(x\right)=\frac{6q\left(x\right)+6p_{n}\left(x\right)+\left(\partial \tau_{t}\left(x\right)+\partial \tau_{b}\left(x\right)\right)h\left(x\right)}{4h\left(x\right)}$$

(26) $$C_{2}\left(x\right)=-\frac{\partial \tau_{b}\left(x\right)-\partial \tau_{t}\left(x\right)}{2h\left(x\right)}$$

(27) $$C_{3}\left(x\right)=-\frac{2q\left(x\right)+2p_{n}\left(x\right)+\left(\partial \tau_{t}\left(x\right)+\partial \tau_{b}\left(x\right)\right)h\left(x\right)}{{\left[h\left(x\right)\right]}^{3}}$$

where

(28) $$\partial \tau_{b}\left(x\right)=\frac{dD_{0}}{dx}+\frac{1}{2}\frac{dD_{1}}{dx}h\left(x\right)+\frac{1}{4}{\frac{dD_{2}}{dx}\left[h\left(x\right)\right]}^{2}$$

(29) $$\partial \tau_{t}\left(x\right)=\frac{dD_{0}}{dx}-\frac{1}{2}\frac{dD_{1}}{dx}h\left(x\right)+\frac{1}{4}{\frac{dD_{2}}{dx}\left[h\left(x\right)\right]}^{2}$$

Stresses calculated at a given point in a chosen cross-section are then substituted in the limit state criterion (1), which provides a local measure of material effort.

## 4. Control Theory Optimization Problem

The analytical model presented above enables the formulation of an optimization problem within the framework of the control theory. The stated problem will be solved with the use of Pontryagin’s maximum principle (PMP), utilizing the direct collocation method in Dircol software.

### 4.1. State and Control Variables and Governing Equations

Here, we introduce the following state variables:
Deflection$X_{1}\left(x\right)=w\left(x\right);$Angle of rotation$X_{2}\left(x\right)=\phi \left(x\right);$Bending moment$X_{3}\left(x\right)=M\left(x\right);$Transverse shear force$X_{4}\left(x\right)=Q\left(x\right);$Beam height$X_{5}\left(x\right)=h\left(x\right);$Derivative of the beam height distribution$X_{6}\left(x\right)=\frac{dh}{dx}.$

The second-order derivative of the beam height distribution will be considered the control variable:

(30) $$U_{1}=\frac{d^{2}h}{dx^{2}}$$

Such an approach is required since the above derivative is required in the determination of the stress tensor components, as can be seen from Equations (23), (28), and (29). Substituting the quantities introduced above in the system of Equation (11), one obtains the following:

(31) $$\left\{\begin{array}{l} X_{1}^{\prime }=X_{2}+\frac{X_{4}}{\kappa GbX_{5}}\\ X_{2}^{\prime }=-\frac{12X_{3}}{Eb{\left(X_{5}\right)}^{3}}\\ X_{3}^{\prime }=X_{4}\\ X_{4}^{\prime }=-q+kbX_{1}\\ X_{5}^{\prime }=X_{6}\\ X_{6}^{\prime }=U_{1} \end{array}\right.$$

### 4.2. Boundary and Compatibility Conditions

The beam is considered to be simply supported; however, the support is modeled by Winkler’s elastic foundation. Boundary conditions should be prescribed for the traction-free end as well as for the middle of the beam, where the symmetry plane is located.

- Traction-free end at x=0:

(32) $$\left\{\begin{array}{c} X_{3}\left(0\right)=0\\ X_{4}\left(0\right)=0 \end{array}\right.$$

- Symmetry condition at x=L/2:

(33) $$\left\{\begin{array}{c} X_{2}\left(L/2\right)=0\\ X_{4}\left(L/2\right)=0 \end{array}\right.$$

Additionally, displacement compatibility conditions and force equilibrium conditions should be prescribed at the end of each characteristic section. At each characteristic point $x_{c}$, the following conditions must be satisfied:

(34) $$\left\{\begin{array}{c} X_{1}\left(x_{c}^{-}\right)=X_{1}\left(x_{c}^{+}\right)\\ X_{2}\left(x_{c}^{-}\right)=X_{2}\left(x_{c}^{+}\right)\\ X_{3}\left(x_{c}^{-}\right)=X_{3}\left(x_{c}^{+}\right)\\ X_{4}\left(x_{c}^{-}\right)=X_{4}\left(x_{c}^{+}\right) \end{array}\right.$$

The final values of the non-prescribed boundary conditions were estimated for the optimal solution in the iterative course of optimization in Dircol software.

### 4.3. Admissible Values of State and Control Variables and Constraints

Constraints on the admissible values of the state and control variables were determined in an iterative way; the following constraints were imposed for each optimization problem:

- PROBLEM 1 (REF beam):

(35) $$X_{5}\in \left(0.38;0.38\right)m,\;\;\;\;\;\;\;\;X_{6}\in \left(-0.25;0.25\right),\;\;\;\;\;\;\;\;U_{1}=\left(-0.25;0.25\right)$$

- PROBLEM 2 ($V_{min}$ beam):

(36) $$X_{5}\in \left(0.25;0.41\right)m,\;\;\;\;\;\;\;\;X_{6}\in \left(-0.25;0.25\right),\;\;\;\;\;\;\;\;U_{1}=\left(-0.25;0.25\right)$$

- PROBLEM 3 ($Q_{max}$ beam):

(37) $$X_{5}\in \left(0.30;0.50\right)m,\;\;\;\;\;\;\;\;X_{6}\in \left(-2.0;2.0\right),\;\;\;\;\;\;\;\;U_{1}=\left(-0.25;0.25\right)$$

Constraints on $X_{6}=\frac{dh}{dx}$ limit the slope of the bottom face of the beam such that simplifications due to the use of the Timoshenko–Ehrenfest theory do not introduce a significant error. Similar constraints on $U_{1}$ limit the curvature of the bottom face. The crucial limitation governing the process of optimization is the constraint related to the limit state condition (1), which corresponds to the ultimate limit state. This condition was checked in every cross-section (for each discretized value of x), as well as for a number of fixed values of the following coordinates: $z_{1}=-0.5\;h,\;{\;z}_{2}=-0.25\;h,\;{\;z}_{3}=0,\;{\;z}_{4}=0.25\;h,and\;z_{5}=0.5\;h$.

The three-dimensional analysis carried out in Abaqus CAE (see Section 4.6), which provided more accurate results, indicated that the values of the stress state components determined using the analytical model were significantly underestimated. This was due to material anisotropy, which was taken into account in the FEM model but disregarded in the simplified beam model. For this reason, the right-hand side of constraint (1) was reduced to 0.85.

Additionally, for each of the three optimization problems, an appropriate constraint was introduced.

- PROBLEM 1 (REF beam):

(38) $$X_{6}=0,\;q=578\frac{kN}{m}$$

- PROBLEM 2 ($V_{min}$ beam):

(39) $$q=578\frac{kN}{m}$$

- PROBLEM 3 ($Q_{max}$ beam):

(40) $$V=V_{ref}$$

### 4.4. Objective Function

The objective function depends on the optimization problem considered.

- PROBLEM 1 (REF beam):

(41) $$J=V=\int_{0}^{L/2}Adx=\int_{0}^{L/2}bX_{5}\left(x\right)dx\to min$$

- PROBLEM 2 ($V_{min}$ beam):

(42) $$J=V=\int_{0}^{L/2}Adx=\int_{0}^{L/2}bX_{5}\left(x\right)dx\to min$$

In problems 1 and 2, the objective function is in the form of an integral function, making it a Lagrange problem. However, the introduction of a new state variable, i.e.,

(43) $$X_{7}\left(x\right)=V\left(x\right)=b\int_{0}^{L/2}X_{5}\left(x\right)dx$$

enables the following objective function:

(44) $$J=V\left(\frac{L}{2}\right)=X_{7}\left(\frac{L}{2}\right)\to min$$

However, an additional state equation must be added to the system governing Equation (31), namely,

(45) $$X_{7}^{\prime }=bX_{5}$$

Thus, the problem is transformed into a Mayer problem.

- PROBLEM 3 ($Q_{max}$ beam):

(46) $$J=-q=X_{8}\to min$$

In problem 3, a new state variable was introduced, i.e., $X_{8}=-q$. In Equation (31), the load density was replaced with the newly introduced state variable. It was also assumed that $X_{8}\left(x\right)=const.$; so, the following state equation was added:

(47) $$X_{8}^{\prime }=0$$

### 4.5. Results of PMP-Based Optimization

After solving problem 1, the minimal height of a beam of constant dimensions was determined to be $h_{min}=38\;cm$. The reference volume is then equal to

(48) $$V_{ref}=bh_{min}L=0.456\;m^{3}$$

The minimal required volume for an optimally shaped beam loaded with a total force of $Q_{ref}=170\;kN$ was found to be equal to

(49) $$V_{min}=0.404\;m^{3}\approx 0.886\;V_{ref}$$

The maximal load that can be applied to an optimally shaped beam of volume $V_{ref}$ was found to be equal to

(50) $$Q_{max}=104\;kN\approx 1.224Q_{ref}$$

**Figure 6:**
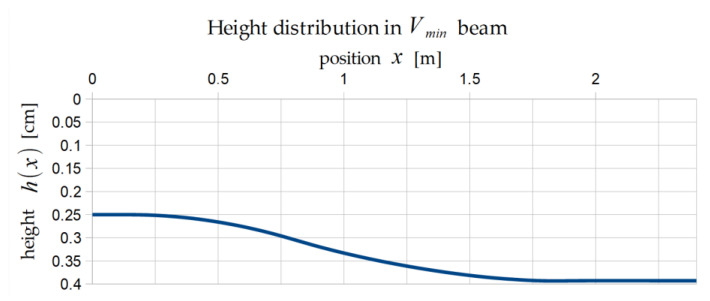
Optimal shape of $V_{min}$ beam (one half).

**Figure 7:**
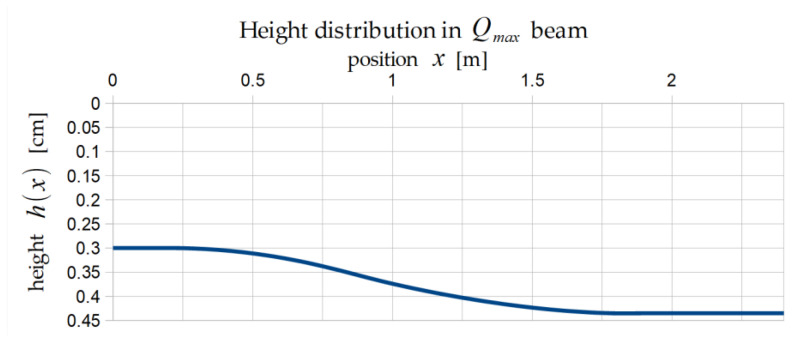
Optimal shape of $Q_{max}$ beam (one half).

The optimal distributions of height are presented below for the volume minimization problem (Figure 6) and the load maximization problem (Figure 7).

**Figure 8:**
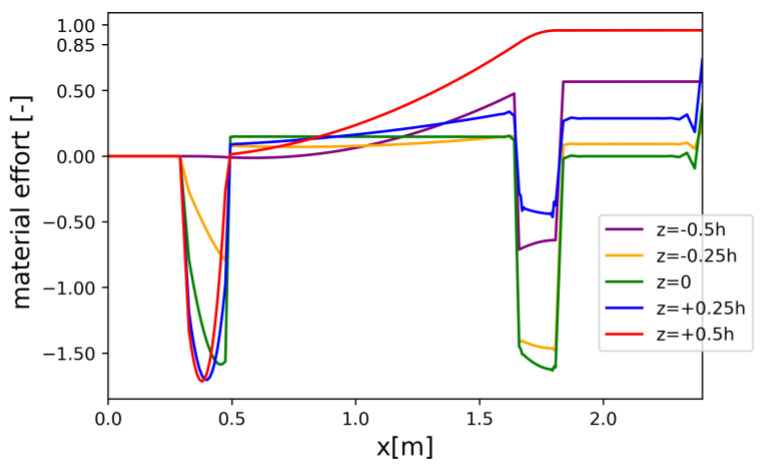
Material effort distribution in REF beam (one half) for chosen z-coordinates.

**Figure 9:**
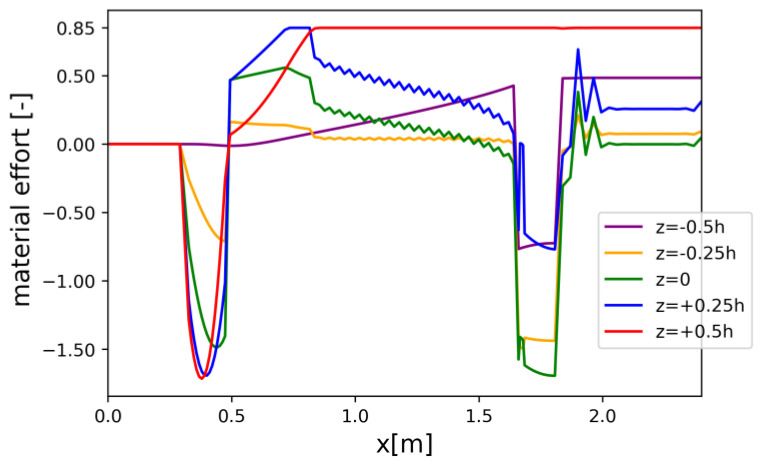
Material effort distribution in $V_{min}$ beam (one half) for chosen z-coordinates.

**Figure 10:**
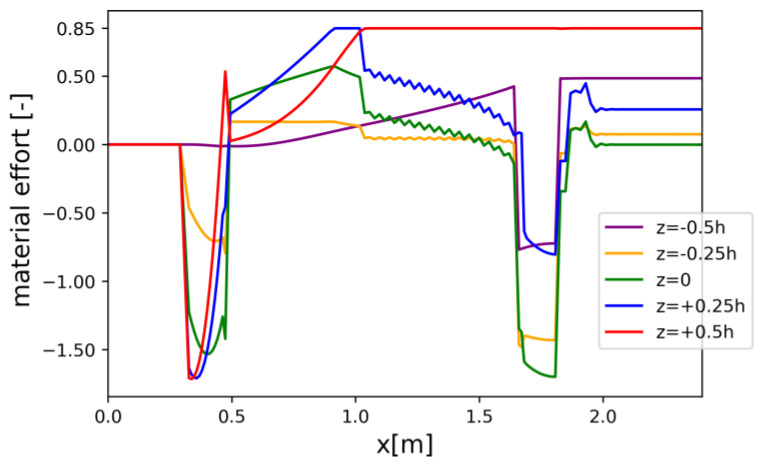
Material effort distribution in $Q_{max}$ beam (one half) for chosen z-coordinates.

Material effort distributions along the beam’s axis for a discrete set of z-coordinate values are presented for the REF beam (Figure 8), $V_{min}$ beam (Figure 9), and $Q_{max}$ beam (Figure 10). Please note that due to oversimplification in the 1D model, which was detected in the course of the 3D FEM analysis, the maximal allowable level of material effort was set to 85% for the *V_min_* and *Q_max_* models.

It was observed that the obtained solution included singular control as the controls appear linearly in the system. The obtained Hamiltonian distribution was approximately piecewise constant. More detailed discussion on singular optimal control problems may be found in [48].

### 4.6. Numerical Validation Adjustment of Optimal Shapes

The beam shapes determined in the optimization task were used to create three-dimensional FEM models designed to replicate the experiment as accurately as possible. Comparative finite element analyses of the beam loading process were conducted using the Abaqus software package.

The GLT was modeled using a linear elastic anisotropic material, with mechanical parameters according to Table 1.

The limit state condition was generalized for the case of a three-dimensional stress state [42]. This formula, under the assumption of zero values for all Poisson ratios, takes the following simple quadratic form:

(51) $$F\left(\sigma \right)=\frac{1}{f_{c,0,k}f_{t,0,k}}\sigma_{xx}^{2}+\frac{f_{c,0,k}-f_{t,0,k}}{f_{c,0,k}f_{t,0,k}}\sigma_{xx}+\frac{1}{f_{c,90,k}f_{t,90,k}}\left(\sigma_{yy}^{2}+\sigma_{zz}^{2}\right)+\;+\frac{f_{c,90,k}-f_{t,90,k}}{f_{t,90,k}f_{c,90,k}}\left(\sigma_{yy}+\sigma_{zz}\right)+\frac{1}{{f_{v,k}}^{2}}\left(\sigma_{xy}^{2}+\sigma_{xz}^{2}+\sigma_{yx}^{2}\right)\leq 1.$$

The FEM mesh consisted of linear brick 8-node elements. A large number of elements (over 200,000) resulted from the need to model the variable shape precisely while keeping the mesh regular to obtain smooth and precise stress maps along the curved bottom surface of the beam as this area experiences the highest material effort.

**Figure 11:**
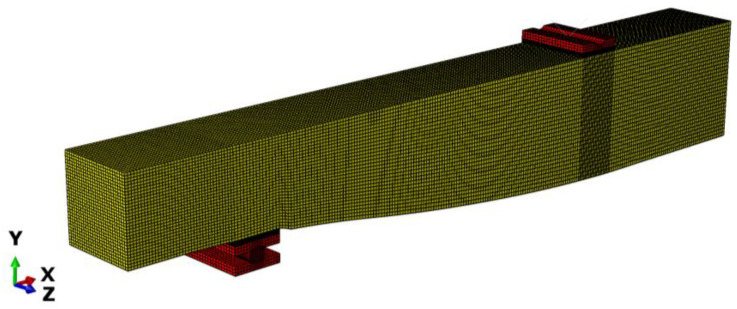
Finite element model and mesh.

The transfer of support and load to the beam was modeled to accurately replicate the experimental setup (see Figure 11). The dimensions of the support components were consistent with the actual values. Rough contact, with a friction coefficient equal to 0.1, was introduced between the components of the steel roller. The beam loading process was simulated as incremental displacement enforcement. The polymer spacers, the stiffness of which was specified in Section 2.2, were modeled above the support and in the load application area. Due to the symmetry of the problem, only half of the girder was considered. The loading process was realized incrementally in the nonlinear static step.

**Figure 12:**
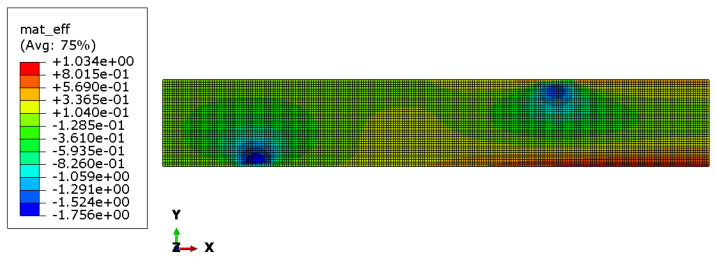
Material effort distribution in REF beam (one half).

**Figure 13:**
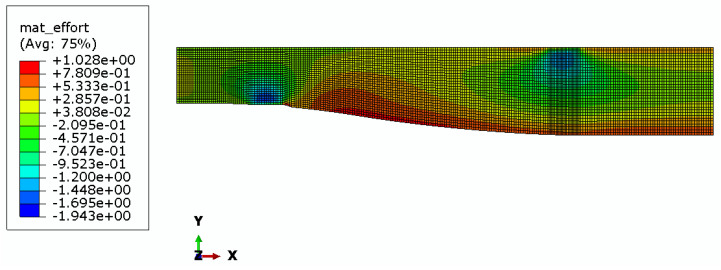
Material effort distribution in $V_{min}$ beam (one half).

**Figure 14:**
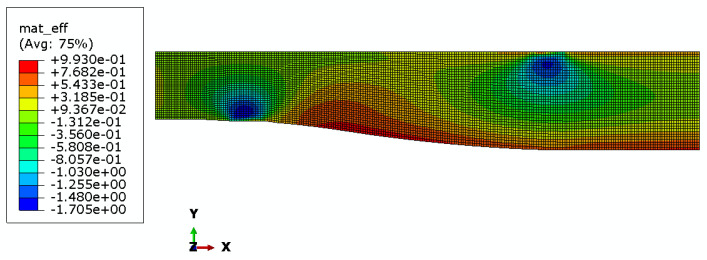
Material effort distribution in $Q_{max}$ beam (one half).

Maps of material effort distribution obtained via the 3D FEA are presented for the REF beam (Figure 12), $V_{min}$ beam (Figure 13), and $Q_{max}$ beam (Figure 14).

## 5. Experimental Validation of Optimization Process

### 5.1. Experimental Setup

Once the optimal beam shapes were determined, they were used as inputs for computerized numerical control (CNC) production. A total of nine beams were manufactured by the KONSBUD company (Stobno 55A, 72-002 Stobno, Poland) from the GL24h-class GLT.

**Figure 15:**
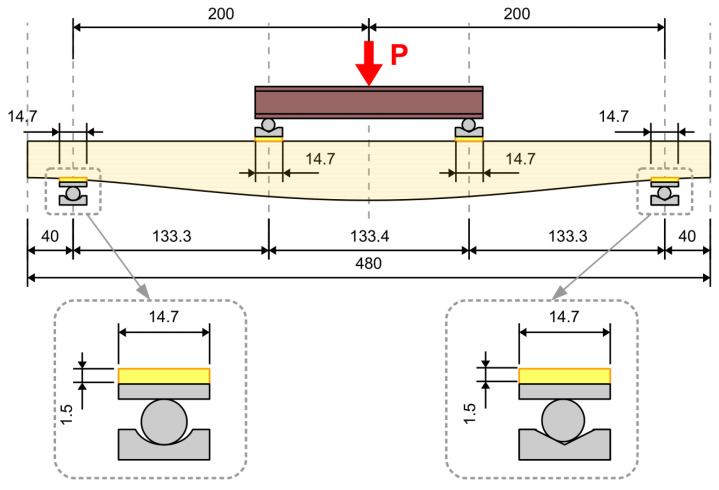
Experimental setup (dimensions in cm).

The beams were simply supported on two steel roller supports with additional polymer spacers that were 147 mm long and 15 mm thick. The horizontal displacement of one of the rollers was constrained. The span length, namely, the distance between bearing axes, was 4 m, while the total length of all the beams was 4.8 m. Overhangs of 40 cm in length were introduced in order to avoid edge effects close to the concentrated load due to supports. The load was applied indirectly through a steel I-section placed on two rollers; in turn, this rested on a steel plate and polymer spacer of the same type as these were used over the supports. The experimental setup is presented in Figure 15.

**Figure 16:**
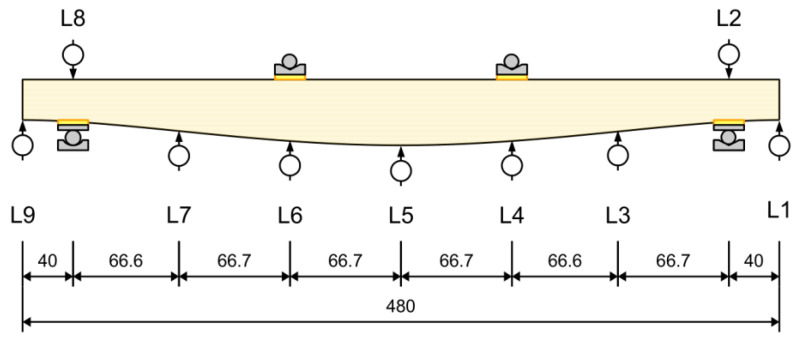
Position of displacement sensors (dimensions in cm).

The vertical deflections of the beams were measured at nine points with the use of inductive displacement sensors. The localization and numbering of the sensors are presented in Figure 16.

**Figure 17:**
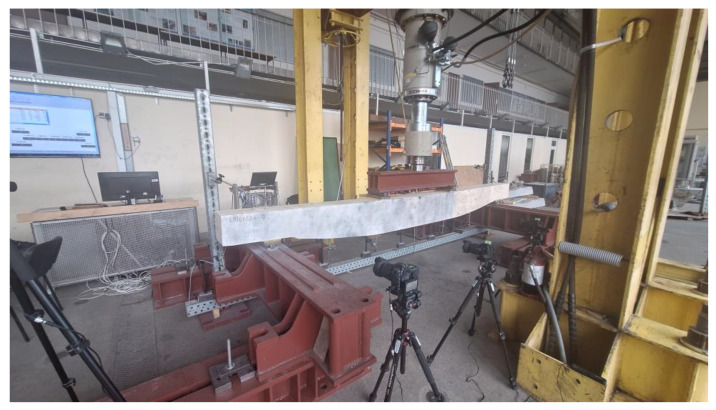
Experimental setup.

The experiment was carried out using an INSTRON Schenk Testing system for force excitation with a Lebtronik 8800 driver (INSTRON, Darmstadt, Germany) (see Figure 17). The load was applied by a PL1000 actuator (INSTRON, Darmstadt, Germany) with a maximal force of up to 1000 kN and a precision class of 0.5. The loading process was displacement-controlled with a uniform displacement rate of 1 mm/min. Displacements in the measurement points L1–L9 were recorded with a Hottinger Baldwin Masstechnik measurement system, utilizing inductive displacement transducers WA/20MM, WA/50MM, and WA100MM, with a resolution of 0.01. A Quantum MX-840B amplifier (HBM, Darmstadt, Germany) with CATMAN EASY AP v. 5.5.1.23 software was used.

**Figure 18:**
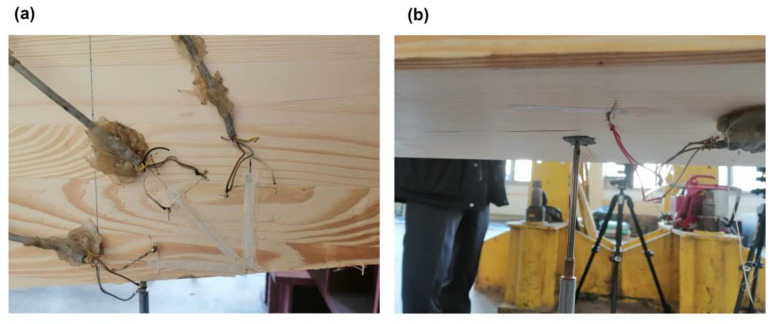
Strain gauges: (**a**) rosette in the area of greatest material effort and (**b**) single-strain gauge on the bottom face in the middle of the span.

Additionally, strain gauge measurements were performed. In the case of the *Q_max_* 3 beam, two electrical resistance strain gauges along the beam’s axis were placed in the middle of the span, one on the top surface and the other on the bottom surface. In the case of all *V_min_* beams, a strain gauge rosette was placed at the side of the beam, close to its bottom surface, 85 cm from the axis of the support, in the area of greatest material effort estimated via FEA (see Figure 13, Figure 14, and Figure 18). Resistance RL 350/50 strain gauges, manufactured by Techno Mechanik (Gdańsk, Poland), were used.

### 5.2. Results

**Figure 19:**
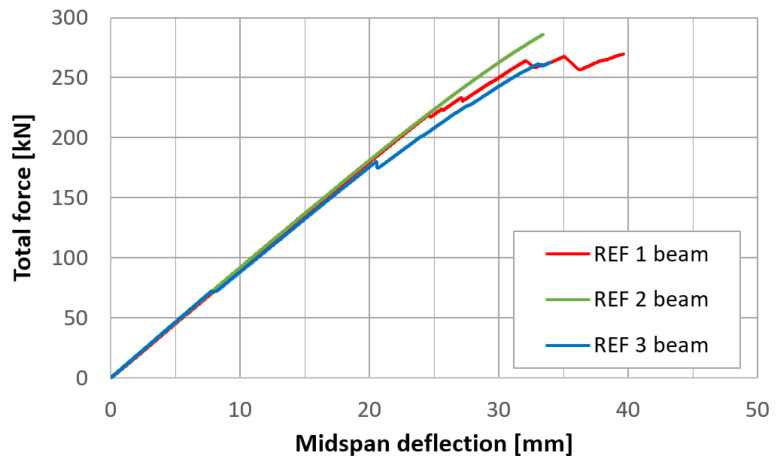
Force–displacement curves for three REF beams.

**Figure 20:**
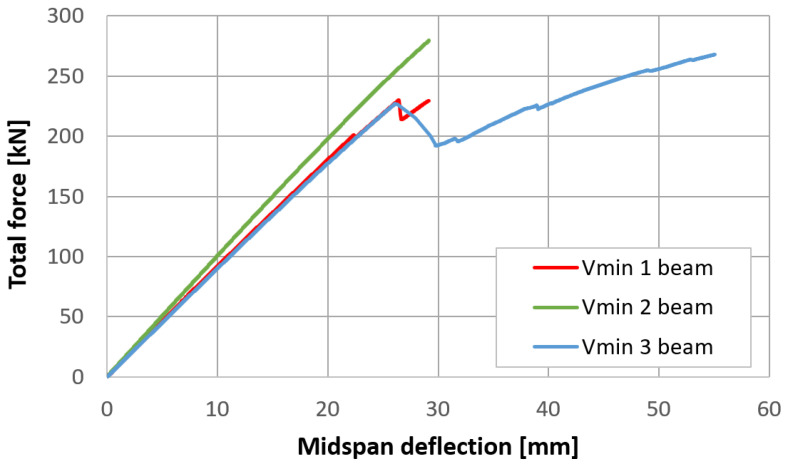
Force–displacement curves for three $V_{min}$ beams.

**Figure 21:**
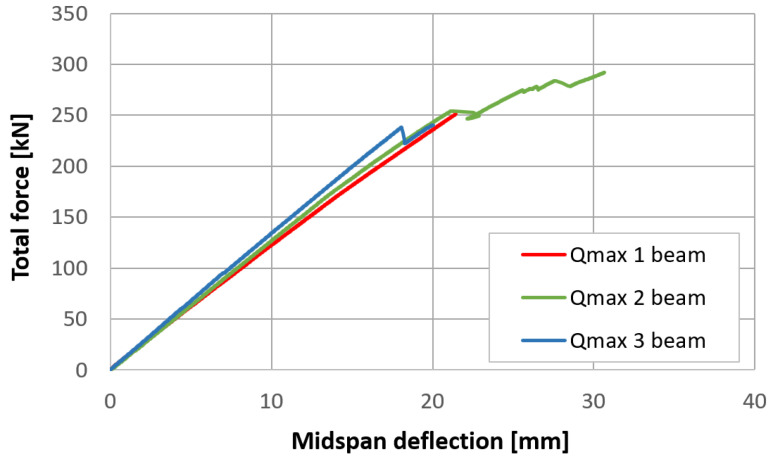
Force–displacement curves for three $Q_{max}$ beams.

Midspan deflections were determined according to the L5 measurement point record, with a correction involving the deformation of elastic spacers over supports, which was an average of the displacements recorded at the L2 and L8 points. Records from the transducers are available in a dataset published with the article. Force–displacement curves for each of the three types of beams are presented in Figure 19, Figure 20 and Figure 21. The endpoint of each curve corresponds to the maximal applied force, beyond which cracking, a sudden drop in force, and an extensive increase in deflection were observed.

Young’s moduli in bending were determined according to the results of the tests carried out on the REF beams. The flexural stiffness k of the beam was calculated as the slope of a linear regression line determined for the section of the force–displacement curve corresponding to the range of force $10\%\;F_{max}÷40\%\;F_{max}$. Young’s modulus was then found according to the stiffness formula for four-point bending, as follows:

(52) $$E=\frac{23}{1296}\frac{kL^{3}}{I}$$

where *I* is the second moment of area of the cross-section. An average value of E=8.99 GPa was obtained, which is significantly lower than the mean value corresponding to the GL24h class (see Table 3).

Flexural stiffness was also determined for the optimized beams. The results are presented in Table 3.

For each beam. three force values were determined: the maximal force, $F_{max}$; the force for which the first significant cracking occurred (the drop in force), $F_{u}$; and the force for which the deviation of the experimental curve from the linear approximation was greater than 5%, $F_{5\%}$. A linear approximation was determined in the same way as the flexural stiffness of the REF beams. The results are summarized in Table 4.

**Figure 22:**
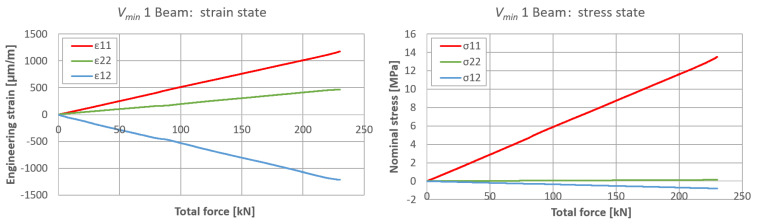
Plane strain and stress tensor components on side surfaces of *V_min_* 1 beam in predicted area of greatest material effort.

**Figure 23:**
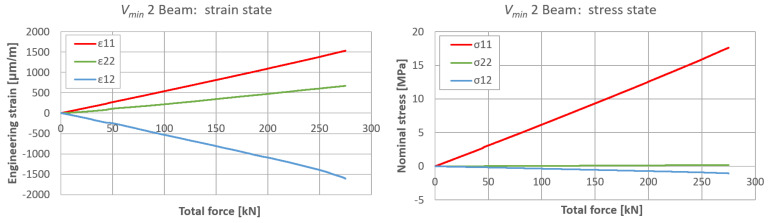
Plane strain and stress tensor components on side surfaces of *V_min_* 2 beam in predicted area of greatest material effort.

**Figure 24:**
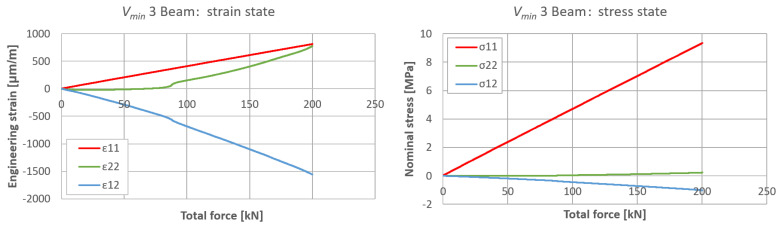
Plane strain and stress tensor components on side surfaces of *V_min_* 3 beam in predicted area of greatest material effort.

**Figure 25:**
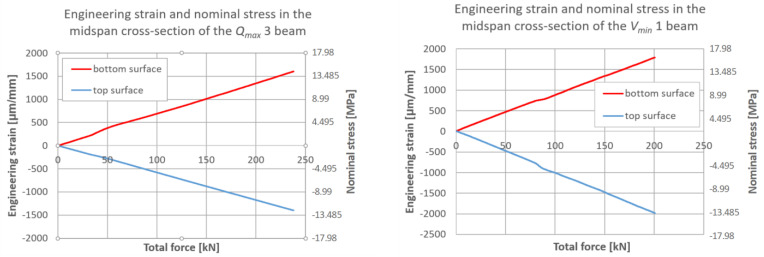
Strain and stress in edge fibers in the middle of the span of optimized beams.

**Figure 26:**
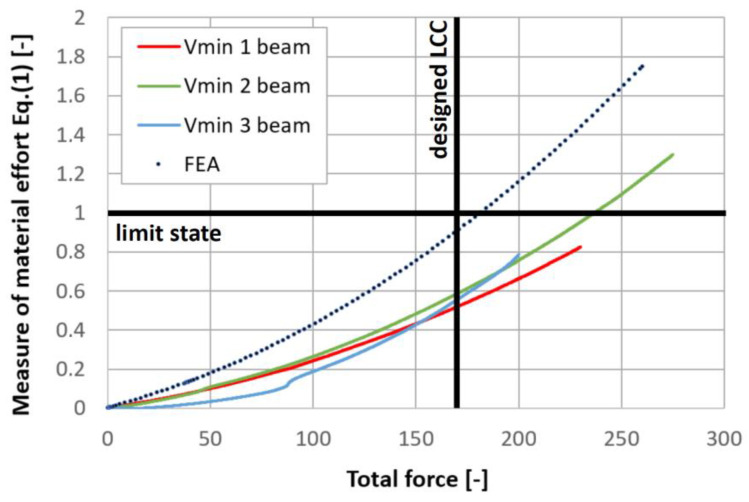
Material effort as a function of applied load according to assumed anisotropic limit state condition. Comparison of strain gauge rosette measurements and FEA predictions.

**Figure 27:**
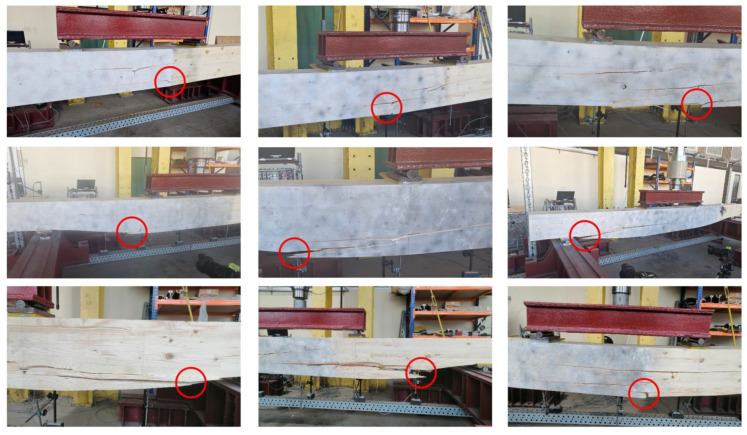
Failure modes of tested beams. Top row: REF beams; middle row: *Q_max_* beams; and bottom row: *V_min_* beams. From left to the right, in all rows, beams are denoted by 1, 2, and 3, respectively. Red circles indicate the areas of failure initiation.

The plane strain state and plane stress state components obtained from the rosette strain gauges are presented in Figure 22, Figure 23 and Figure 24 for each *V_min_* beam. The engineering strains and nominal stresses in the top and bottom fibers of the midspan cross-section in the optimized beams *Q_max_* 3 and *V_min_* 2 are presented in Figure 25. A graph illustrating the increase in material effort according to the strain gauge rosette measurements is presented in Figure 26. FEA predictions are also plotted for comparison. The results from the numerical simulations were obtained using the 3D version of the limit state condition; however, it differed from the 2D variant by less than 1%. Please note that the values of the stress tensor components, as well as measurements of material effort, were calculated based on the mean values of the stiffness moduli given in Table 1. The failure modes of the examined beams are presented in Figure 27.

The magnitudes of the stress tensor components corresponding to the designed LCC obtained from the strain gauge measurements, as well as those predicted by the FEA for *V_min_* beams, are listed in Table 5.

## 6. Discussion

Two optimization problems were stated for simply supported GLT beams under four-point bending: minimizing the volume for a given LCC and maximizing the LCC for a given volume of material. The mechanical properties of the material were assumed according to the Eurocode regulations regarding the GLT class provided by the manufacturer. An energy-based anisotropic limit state criterion was used for the analysis of complex stress states. A control theory problem was formulated and solved by applying Pontryagin’s minimum principle with the use of a direct collocation method implemented in Dircol software. A scalar singular problem, which is significantly more difficult than regular non-singular problems, was solved. The obtained optimal height distribution was then used to develop a detailed 3D FEM model. The analysis performed indicated that the effect of anisotropy necessitates more restrictive limit state conditions in certain parts of optimized beams. After introducing this correction, PMP-based optimization and FEA were performed again. Three types of nine GLT beams were manufactured and tested.

Significant variation in the results may be observed due to the sensitivity of the timber elements’ ultimate load-carrying capacity to local defects, such as knots or cracks. It can be observed that the performance of the $V_{min}$ 2 beam differs significantly from the behavior of the remaining specimens of the same kind; for this reason, the results obtained for this beam could be considered outliers. For such a small number of specimens, however, one cannot accurately predict the mean values and their ranges in order to determine if this result does indeed deviate from the expected response of beams belonging to a much larger population. In order to arrive at an unambiguous conclusion, a much larger statistical sample should be examined.

It should also be noted that the true total load-carrying capacity of the manufactured beams was approximately 55% greater than the designed one, equal to 170 kN; it is approximately 39% greater than the mean $F_{u}$, and this is similar for $F_{5\%}$. This is because the design strength values employed in the calculations correspond to the 5% quantile; so, there was a 95% probability of observing a higher load-carrying capacity. Another issue is that the timber’s strength is most often classified according to visual inspection, i.e., not according to statistical considerations. While this simplified procedure is often considered safer, it is usually not possible to acquire more accurate data from the timber provider; so, designers do not have the opportunity to achieve precise structural optimization.

- An interesting observation is that the $F_{u}$ and $F_{5\%}$ of REF beams are clearly lower than those of optimized beams. However, in our opinion, this might be unrelated to the optimization problem since both the first instance of cracking and the linearity of the beam’s response are highly affected by local flaws in the material.
- The rectangular REF beams all failed in the middle of the span because the tensile strength of the fibers on the bottom face of the beam was exceeded. The crack pattern indicates that the shear strength was also exceeded in the areas under applied load near the neutral axis, which is the area of maximal shear stress. Almost all optimized beams (except for the *V_min_* 3 beam) were destroyed due to failure in the predicted area of material effort concentration (see Figure 13 and Figure 14). Shear failure along the wood grain may be observed; however, crack initiation occurred due to a multidimensional stress state at the oblique boundary involving both shear stress and transverse normal stress. The material strength values for these two stress components are the smallest of all the strength parameters (see Table 1). The fact that the *V_min_* 3 beam was destroyed in a similar manner as the REF beams may indicate that optimization was successful, namely, that the beams were in an approximately fully stressed state.

## 7. Conclusions

Large variations in the mechanical properties of wood, insufficient information on the mechanical characteristics of specific elements, and the relatively small statistical sample make it impossible to formulate strict conclusions. Nevertheless, the following inferences may be drawn:

- Reference beams with a constant cross-section height exhibit a larger load-carrying capacity due to the redundant amount of material, which provides overall cohesion even after crack initialization. It may be noted that the average load required to cause the first instance of cracking in the REF beams was the smallest of all the examined beam types, while the maximal applicable load was the largest.
- The overall performance of both optimized beam types (*V_min_* and *Q_max_*) was very similar. This is probably due to local defects in the wood and the insufficient size of the statistical sample; the *Q_max_* beams did not exhibit a significantly larger load-carrying capacity.
- Practically all cracks in the examined beams propagated through the GLT without aligning with the lamella interfaces. This indicates that the adhesive layers bonding the lamellae in the GLT do not introduce any significant inhomogeneity in the mechanical and strength properties of the GLT.
- The optimized beams exhibited significantly larger flexural stiffness, which is particularly evident in the *Q_max_* specimens compared to the REF beams. While both beam types had the same volume, $V_{ref}$, in the case of the optimized beams, larger cross-sections were found in the areas of the greatest bending moments (due to the optimization of the load-carrying capacity), which simultaneously contributed to the decrease in the local curvature of the deformed beams and, consequently, to the smaller deflections.
- Strain gauge measurements indicate that the normal stress in the edge fibers in the middle of the span in the optimized beams, corresponding to the smallest force causing initial cracking (not in the midspan cross-section), is smaller than the characteristic tensile or compressive strength. However, in the case of the optimal *V_min_* 1 beam, the tensile stress, corresponding to $F_{u}=201\;kN$ (compared to $F_{max}=230\;kN$), is close to $f_{t,0,k}$.
- Stress tensor components and material effort were estimated using the mean values of the stiffness moduli for the assumed class of the GLT and records from strain gauge rosettes. It can be observed that while the longitudinal normal stress was predicted correctly by the FEA, the numerical estimates of $\sigma_{zz}$ and $\sigma_{xz}$ were approximately twice the measured values. Since these two components influence the limit state condition to the greatest extent, the measure of material effort also seems to be overestimated. Finding the accurate magnitudes of stress and the material effort according to the measurements performed using rosette strain gauges is impossible without knowing all the true elastic constants of the GLT. This is the subject of ongoing research, including material testing on destroyed GLT beams and the comparison of the strain gauge records with full-field DIC measurements.

The scope of applicability of the proposed method is limited in the case of the GLT because it is impossible to take into account the anisotropy of the material in a 1D beam model. Transverse normal stresses and shear stresses influence the proposed measure of material effort to a significant extent, even for small values of these stresses. These two components of the stress tensor must be fairly estimated, which cannot be accomplished with the use of a simplified model; more accurate finite element analyses must be performed in such cases.

Further limitations of the potential use of the presented approach are related to modeling a solid element with a 1D beam. Stress concentration due to the presence of notches along the beam’s axis and the contour of the cross-section is necessarily neglected. Significant curvatures and large slopes of the bottom face of the beam also cannot be modeled appropriately with the use of a 1D beam model since the boundary conditions on the oblique face are taken into account only in an approximate way. It should be emphasized that local equilibrium equations are also formally violated, which may lead to significant errors in the case of large variations in beam height along its axis.

This research indicates that material savings are possible when designing GLT beams. It should be noted, however, that due to the characteristics of CNC cutting when using beams of variable height, these savings would be primarily in the form of scrap wood, which can be utilized in the production of other wood-based products. Optimization via Pontryagin’s maximum principle has emerged as a useful tool, although it must be enhanced with additional analyses that take into account the full three-dimensional stress state to determine the optimal shape with sufficient accuracy.

While the present investigation focused on the possibility of optimizing shape using available design data (characteristic strengths and mean stiffness moduli), our future research aims to compare predicted stress and strain states to those determined via full-field digital image correlation (DIC) measurements. Displacement maps have already been recorded (speckle patterns may be observed in Figure 17 and Figure 27); however, in order to find the corresponding stress state, one needs to know the actual elastic constants instead of the characteristic ones. Standard compression tests are intended to be performed on pieces cut out from the undamaged parts of the investigated beam in order to estimate the true Young’s modulus in two directions, the shear modulus, and Poisson’s ratio. DIC measurements will also be employed to determine elastic constants.

## Figures and Tables

**Table 1:** Mechanical properties of GL24h-class GLT according to EN 14080:2013 [38].

Mechanical Property	Value
Characteristic flexural strength $f_{m,k}$	24 MPa
Characteristic tensile strength parallel to grain $f_{t,0,k}$	19.2 MPa
Characteristic tensile strength perpendicular to grain $f_{t,90,k}$	0.5 MPa
Characteristic compressive strength parallel to grain $f_{c,0,k}$	24 MPa
Characteristic compressive strength perpendicular to grain $f_{c,90,k}$	2.5 MPa
Characteristic shear strength $f_{v,k}$	3.5 MPa
Mean modulus of elasticity parallel to grain $E_{0,m}$	11.5 GPa
Characteristic modulus of elasticity parallel to grain $E_{0,k}$	9.6 GPa
Mean modulus of elasticity perpendicular to grain $E_{90,m}$	0.3 GPa
Mean shear modulus $G_{m}$	0.65 GPa
Poisson ratio ν (according to [39])	0

**Table 2:** Parameters of limit state criterion for GL24h-class GLT.

Parameter
$A_{I}=0.0021701\;{\left(MPa\right)}^{-2}$
$A_{II}=0.8000000\;{\left(MPa\right)}^{-2}$
$A_{III}=0.0816327\;{\left(MPa\right)}^{-2}$
$B_{I}=0.0104167\;{\left(MPa\right)}^{-1}$
$B_{II}=1.6000000\;{\left(MPa\right)}^{-1}$

**Table 3:** Flexural rigidity of beams in kN/m.

Beam type	Beam 1	Beam 2	Beam 3	Average	Std. Dev.
REF	9022.337	9187.666	8947.826	9052.610	122.75
$V_{min}$	9208.991	10,082.187	8999.455	9430.211	574.27
$Q_{max}$	12,391.280	12,662.917	13,603.633	12,885.943	636.20

**Table 4:** Forces characterizing the flexural performance of beams.

Beam type	Specimen	$F_{max}$ [kN]	$F_{u}$ [kN]	$F_{5\%}$ [kN]
REF	REF 1	269	219	232
	REF 2	286	286	266
	REF 3	263	180	175
Mean REF		273	228	224
Std. Dev. REF		11.93	53.61	45.98
V_min_	V_min_ 1	230	201	220
	V_min_ 2	280	280	280
	V_min_ 3	268	227	227
Mean V_min_		259	236	242
Std. Dev. V_min_		25.80	40.09	32.60
Q_max_	Q_max_ 1	251	251	239
	Q_max_ 2	291	251	251
	Q_max_ 3	240	238	232
Mean Q_max_		261	247	241
Std. Dev. Q_max_		26.84	7.51	9.61

**Table 5:** Stress tensor components in the area of predicted concentration of material effort in Vmin beams—data recorded by strain gauge rosettes and FEA predictions for P = 170 kN.

	FEA	*V_min_* 1 Beam	*V_min_* 2 Beam	*V_min_* 3 Beam
$\sigma_{xx}\;[MPa]$	9.67	9.89	10.6	7.96
$\sigma_{zz}\;[MPa]$	0.235	0.104	0.119	0.162
$\left|\sigma_{xz}|\;[MPa\right]$	1.53	0.589	0.598	0.829
material effort Equation (1)	0.916	0.519	0.586	0.556
$\sigma_{yy}\;[MPa]$	−2.8 × 10^−5^			
$\left|\sigma_{xy}|\;[MPa\right]$	2.3 × 10^−4^			
$\left|\sigma_{zy}|\;[MPa\right]$	2.7 × 10^−4^			
material effort Equation (1)	0.915			

## Data Availability

All data may be shared upon request to the corresponding author.
